# Development of a High Flow Rate 3-D Electroosmotic Flow Pump

**DOI:** 10.3390/mi10020112

**Published:** 2019-02-11

**Authors:** Zi Ye, Renchang Zhang, Meng Gao, Zhongshan Deng, Lin Gui

**Affiliations:** 1 Key Laboratory of Cryogenics, Technical Institute of Physics and Chemistry, Chinese Academy of Sciences, 29 Zhongguancun East Road, Haidu District, Beijing 10019, China; yezi15@mails.ucas.ac.cn (Z.Y.); zhangrenchang15@mails.ucas.ac.cn (R.Z.); mgao@mail.ipc.ac.cn (M.G.); zsdeng@mail.ipc.ac.cn (Z.D.); 2 School of Future Technology, University of Chinese Academy of Sciences, 19 Yuquan road, Shijingshan District, Beijing 100039, China

**Keywords:** multi-layer structure, electroosmotic flow (EOF) pump, parallel fluid channels, liquid metal electrodes

## Abstract

A low voltage 3D parallel electroosmotic flow (EOF) pump composed of two electrode layers and a fluid layer is proposed in this work. The fluid layer contains twenty parallel fluid channels and is set at the middle of the two electrode layers. The distance between fluid and electrode channels was controlled to be under 45 μm, to reduce the driving voltage. Room temperature liquid metal was directly injected into the electrode channels by syringe to form non-contact electrodes. Deionized (DI) water with fluorescent particles was used to test the pumping performance of this EOF pump. According to the experimental results, a flow rate of 5.69 nL/min was reached at a driving voltage of 2 V. The size of this pump is small, and it shows a great potential for implanted applications. This structure could be easily expanded for more parallel fluid channels and larger flow rate.

## 1. Introduction

Micropumps are one of the essential components in microfluidics systems [1]. The applications of micropumps include biological analysis [2,3,4], drug delivery [5], and micro-mixers [6]. For all these applications, micropumps are required to have wide range of flow rates, stable flow and biocompatibility. Electroosmotic flow (EOF) pumps have drawn much attention in recent years. They have the ability to generate constant flows, precisely control of flow rate, and can be easily integrated into lab-on-a-chip system because they have no moving parts [7]. Thus, they could offer a great solution to liquid delivery in microfluidics.

According to fluid channel type, EOF pumps can be divided into two categories: porous channel pumps and direct channel pumps. Porous channel EOF pumps could generate high flow rates at a relatively low voltage [8,9,10]. In some designs, particles with a diameter of a few microns are added into capillaries to form the fluid channel [8]. Also, both the porous membrane [9] and porous monolith [10] are used as fluid channels, and the flow rate of several mL/min would reach under 100 V. The fabrication process of these pumps is complicated, and pore diameter limits the sample size in the fluid. Cells and large particles are unable to go through these kinds of channels. This greatly limits their usage in biological analysis. On the other hand, direct channel EOF pumps have nothing blocking the channel, and could expand the usage of the pumps. One kind of direct channel EOF pump uses capillary as fluid channels [11]. Voltage as high as several thousand volts is applied directly on both sides of the capillary. The flow rate would reach several μL/min. As micro fabrication develops, materials such as polydimethylsiloxane (PDMS) and polymethyl methacrylate (PMMA) are used to fabricate EOF pumps [12]. Solid electrodes are integrated in PDMS, and directly contact the microfluidic channel. Compared to single channel EOF pumps, parallel fluid channels could enhance the flow rate. With 10 parallel channels, flow rate would be increased compared to single channel pumps [13]. The electrodes in most of these pumps contact the fluid directly while pumping, and this would cause contamination to the fluid samples. Strong electrophoresis would happen with contact electrodes. Moreover, while working, bubbles and Joule heat would be a limitation of flow rates [14] and applications to these pumps.

To improve the bubble and joule heat problems in direct channel EOF pumps, and to reduce electrophoresis, several solutions are proposed: alternative current (AC) EOF pumps [15,16], bubbleless electrodes EOF pumps [17] and non-contact EOF pumps [1,18]. AC EOF pumps use asymmetric electrodes. Bubbleless electrodes such as vinylized fused silica capillary electrodes require high cost and complicated fabrication process. Non-contact EOF pumps, whose electrodes are not in direct contact with fluid channel, are ideal for EOF pumps. Room temperature liquid metal (gallium-base alloy) is used as electrodes and this EOF pump is able to generate fluid flow at a voltage less than 2 V [1]. Later, solid gallium metal electrodes were also used in non-contact EOF pumps in a similar pump structure, and the lowest pumping voltage is 650 V [18]. However, the flow rates of these pumps are relatively low, only several nanoliters per minute. In these 2-dementional designs, there could be only one or two fluid pumping channels. It is very difficult to increase the flow rate by using parallel fluid channel without increasing the pump size too much. To increase the flow rate, 3-dementional structure EOF micropump could be considered.

In this work, a low voltage three-dimensional non-contact EOF pump with new 3D structure is proposed. The pump is made of PDMS, and gallium-based alloy is used to form electrodes. This type of alloy is in liquid form in room temperature, and can be injected into channels directly by syringe. This alloy is also widely used as micro-electrode [19], biomaterial [20] and 3D printing material [21]. The structure and fabrication process of this pump is introduced below in detail. Then, we describe its performance, which we tested using deionized (DI) water with fluorescent particles for flow tracing. Experiment results are shown and discussed at the end of this paper.

## 2. Materials and Methods

### 2.1. Design of 3-Dimentional Non-Contact EOF Pump

**Figure 1:**
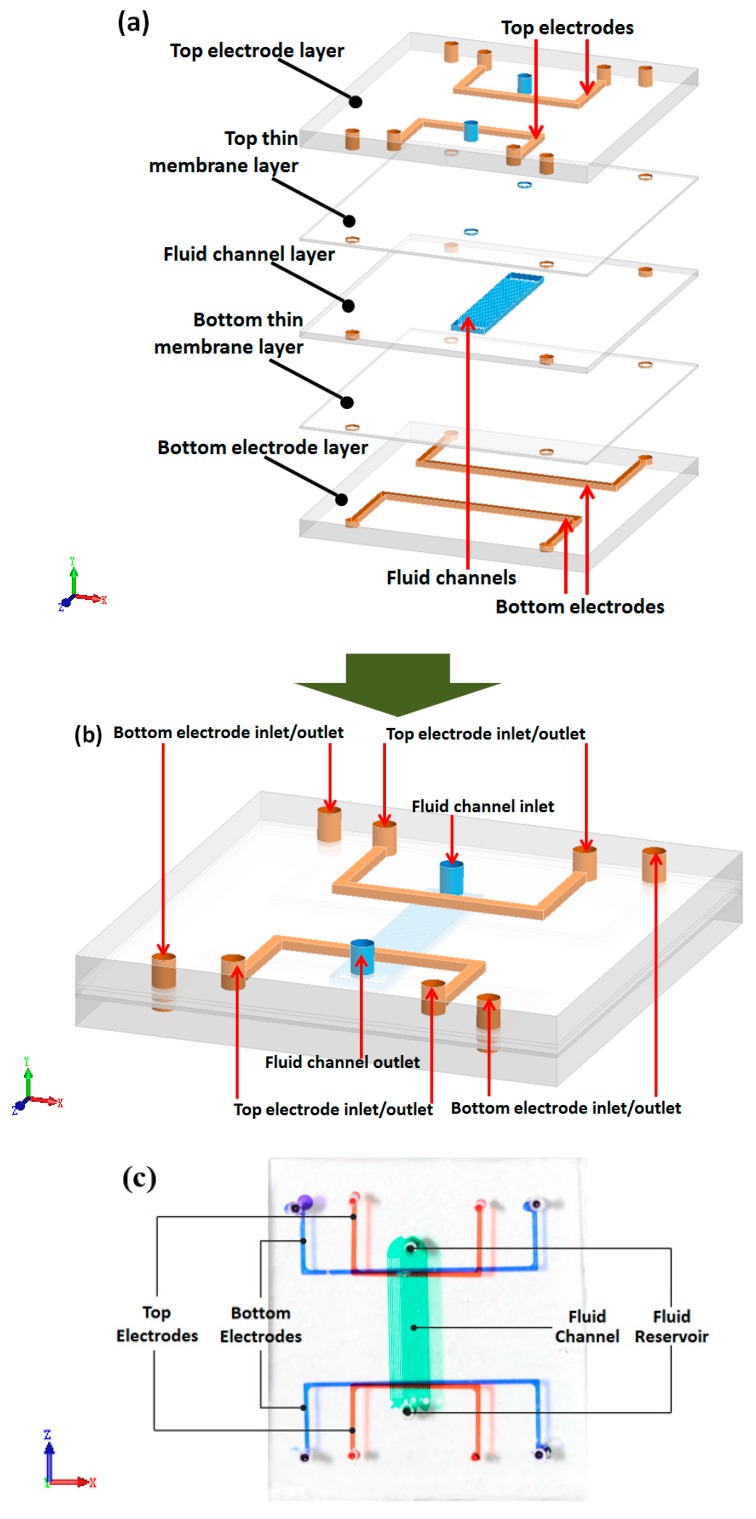
Schematic of the electroosmotic flow (EOF) pump. (**a**) Layer view; (**b**) combination view; (**c**) optical photograph of 3-demintional EOF pumps.

Figure 1a,b shows the schematic of this stereoscopic pump. It contains 5 layers, including three channel layers and two thin membrane layers. The three channel layers contain two electrode layers and one fluid layer. The fluid layer lies in the middle of two electrode layers. Between the fluid layer and both electrode layers there are two thin membrane layers. These two thin membrane layers make the electrode not in direct contact with working fluid. 

**Figure 2:**
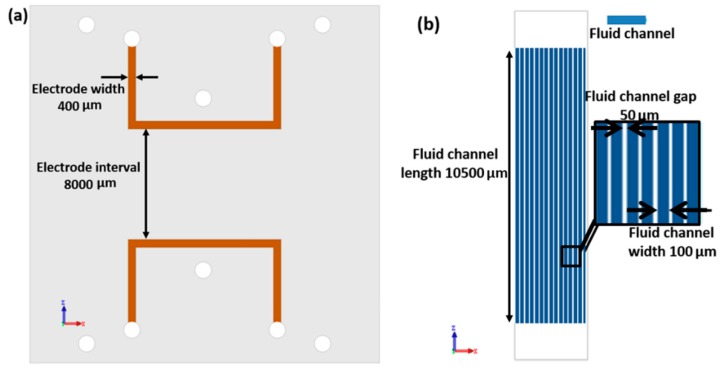
Schematic of the dimensions of EOF pump. (**a**) Electrode channel (top view) (**b)** fluid channel (top view).

All three channel layers were designed separately. Each of the two electrode layers contains two symmetric electrode channels. For the convenience of injecting liquid metal, electrode channels were designed in a U shape, with one injection inlet and outlet. The width of those channels is 400 μm, consistently. The distance between electrodes on the same layer is 8000 μm. Fluid channels are set perpendicular to the electrodes. In this work, we use 20 parallel channels as fluid channels. All 20 channels share the same inlet and outlet. The width of these fluid channels is 116 μm each, and the gap between the adjacent channels is 50 μm. The length of fluid channel is 10500 μm. Moreover, in order to reduce the driving voltage of the pump, the membrane layers are made as thin as possible. Due to different fabrication process, the bottom thin membrane is 16μm thick, and top membrane is 43 μm thick. The dimension schematic of this pump is shown in Figure 2.

**Figure 3:**
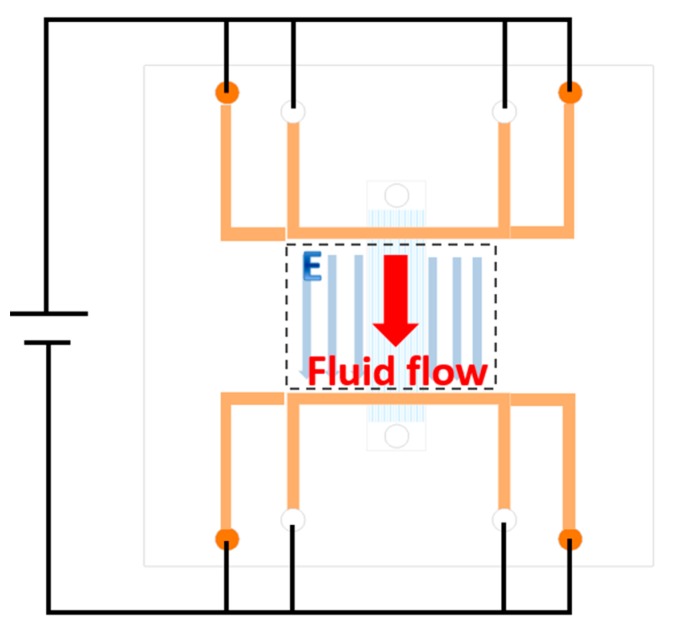
Working principle.

While working, a voltage is applied between two electrodes on both top and bottom electrode layers, as shown in Figure 3. Because the U shapes of the electrode, there are two long parallel electrodes on both sides of fluid channels. Thus, there is a uniform electronic field along the fluid channels. Electroosmotic flow would then be generated in response to the electrode field.

With the design including parallel fluid channels that are not in the same layer as the electrode channels, the number of fluid channels could be easily expanded if needed. Flow rate can be increased by making more parallel fluid channels. The distance between fluid channels and electrode channels is controlled to be under 45 μm, and this lowers the driving voltage applied to the pump. Non-contact electrode design would prevent the bubble generation due to the electrode corrosion, and increase the stability and lifetime for this pump. Also, sample contamination would be prevented for biological applications.

### 2.2. Fabrication Process

The material of the chip is PDMS, and the electrode material is gallium-based alloy (Ga_66_In_20.5_Sn_13.5_). The first step of making this pump is to prepare patterned or empty PDMS. All the channels are made by standard soft photolithography. Both top and bottom electrode layers are made by directly pouring PDMS on patterned silicon wafers. In the real fabrication process we found it difficult to make the PDMS membrane exactly as thin as the channel height. So, we combined the top thin membrane layer with fluid channel layer in fabrication process. This combined layer is made by spinning PDMS on a silicon wafer with fluid channel pattern at 1000 RPM, to form a 74 μm thick membrane. With 31 μm high channels, the membrane above fluid channel is 43 μm thick. The bottom thin membrane layer is made in the same way, except an empty silicon wafer is used and the speed is 3000 RPM, resulting in a 16 μm thick membrane. The four layers are then baked on the hot plate to solidify PDMS. 

The second step is to remove PDMS and to bond each layer. In this step, we found it hard to remove a thin PDMS membrane from a silicon wafer without damaging it. Our solution is to bond a thick PDMS block on a thin membrane and remove the two layers together. In this case, we first bond the thick top electrode layer to fluid channel layer and remove the two layers together. Then, first two layers are bonded to the bottom thin membrane layer, and finally to the bottom electrode layer. The bonding process used oxygen plasma treatment. It is worth mentioning that the electrode channels of the top and bottom layers should be aligned preciously to guarantee the homogeneity of electric fields in the fluid channel. An align machine (Wenhao, Suzhou, China) was used to do the alignment. There are two aligning platforms in the machine. Two PDMS layers were stuck on these two platforms respectively and made the alignment under a microscope. Then these two platforms were taken off from the machine together with the PDMS layers and put into the plasma cleaner (Yanzhao Technology, Tangshan, China) to make the plasma treatment. Finally, the platforms were taken out quickly and put back to the align machine to make the final bonding of the two PDMS layers.

After finishing the four-layer structure, gallium-based alloy is injected into the electrode channels to form electrodes. Copper lines are used to connect electrode with power source. Package adhesive sealant is used to seal the copper wire and liquid metal, to fasten the connection. Figure 1c shows an optical photograph of the EOF pump. The fabrication process of this pump is simple and low-cost.

### 2.3. Experiment

The performance of this high flow rate 3-D EOF pump was tested in experiments. The working fluid was deionized (DI) water. Fluorescent particles with 0.52 μm diameter were added into the DI water by 1:10000 to test the flow rate. The fluorescent particles were 1% solid Red Fluorescent Polymer Microspheres from Fluoro-Max^TM^ (Thermo Scientific, Waltham, MA, USA). The excitation maxima of this particle is 542 nm, and the emission maxima is 612 nm. Zeiss Observer.Z1 microscope (Oberkochen, Germany) and X-cite Series 120Q (Excelitas Technologies, Waltham, MA, USA) laser source were used to observe particle movement. In the experiment, we used a large droplet to cover both the inlet and the outlet of the fluid channel. After putting a large droplet on the inlet and the outlet, the pressure-driven flow was balanced quickly in just several seconds. In each experiment, before applying voltage we made sure all the fluorescent particles were stagnant in the microchannel. Thus, pressure-driven flow can be neglected, and the fluid flow we observed was only EOF.

## 3. Results and Discussion

While measuring electroosmotic flow (EOF) velocity with fluorescent particles, electrophoresis (EPH) velocity needs to be considered. According to Ref. [22], measured velocity can be calculated by following equation:

(1) $$u_{\mathrm{meaure}}=u_{\mathrm{eof}}+u_{\mathrm{eph}}+u_{\mathrm{pressure}}$$

(2) $$u_{\mathrm{eph}}=M_{\mathrm{eph}}\;\times E$$

where $u_{\mathrm{eof}}$ is the flow velocity of EOF, $u_{\mathrm{eph}}$ is the flow velocity of EPH, $u_{\mathrm{pressure}}$ is velocity of pressure-driven flow,$\;M_{\mathrm{eph}}$ is EPH mobility, and E is electric field strength. In this experiment, pressure-driven flow can be neglected. In order to estimate $M_{\mathrm{eph}}$, an EPH experiment was performed. The same particles with same concentration was used in this electrophoresis experiment. DI water with particles filled a large, round PDMS reservoir (*r* = 0.8 cm, *h* = 0.5 cm). Two platinum electrodes were put respectively at each end of the diameter of the reservoir. The diameter of the Pt electrode was 0.5 mm, so the distance between two electrodes was 0.7 cm. A 10 V voltage was added between the two electrodes. Particles moved towards and finally gathered at the positive electrode, proving that the particles are negatively charged. During the experiments, 10 particles moving directly from cathode to anode were chosen to estimate $M_{\mathrm{eph}}$. Experimental results showed that $M_{\mathrm{eph}}=-5.49\;\mu m\;\mathrm{cm}/V\;s$ according to Equation (2).

**Figure 4:**
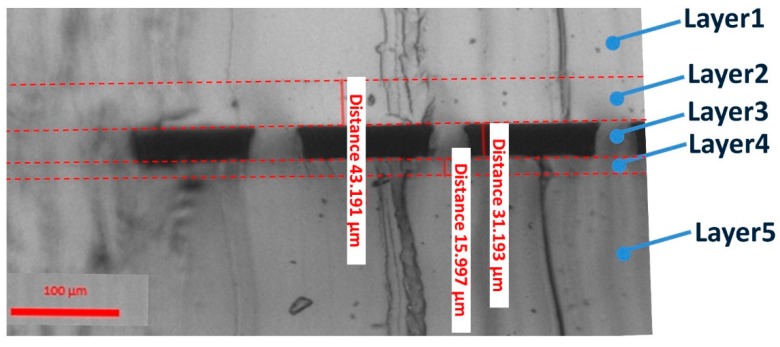
Side view of real pump chip dimensions. Layer 1: top electrode layer (electrode channel not shown here); layer 2: top membrane layer; layer 3: fluid channel layer; layer 4: bottom thin membrane layer; layer 5: bottom electrode layer (electrode channel not shown).

In order to get the electric field strength in the experiment, an electric field simulation of this pump was performed with commercial software Comsol 5.2 (COMSOL Inc., Stockholm, Sweden). One fluid channel, two pairs of electrodes and a PDMS block are set in the model. All geometric parameters used in the simulation model are the parameters we measured in the real pump, as shown in Figure 4. The dielectric constant of PDMS we used (SYLGARD 184 Silicone Elastomer Kit, Dow Corning, MI, USA) was found on Dow Corning’s official website (https://consumer.dow.com/) with a value of 2.72. The dielectric constant of water and liquid metal are set according to well-known values. Thus, the electric field strength in the fluid channel is simulated. For the driving voltage of 10 V, the simulated electric field strength was 0.205 V/cm at the point where we measured flow rate and velocity with fluorescent particles. Then, at 10 V, $u_{\mathrm{eph}}$ is calculated to be $-6.753\times 10^{-2}\;\mathrm{mm}/\min$ based on Equation (2). According to the experiment results, $u_{\mathrm{meaure}}$ at 10 V is $3.60\times 10^{-1}\;\mathrm{mm}/\min$. So, $u_{\mathrm{eof}}\;\mathrm{is}\;4.28\times 10^{-1}\;\mathrm{mm}/\min$ based on Equation (1). The ratio of EOF velocity and measured velocity is 1.188. This result will be used in the following experiment result calculation.

**Figure 5:**
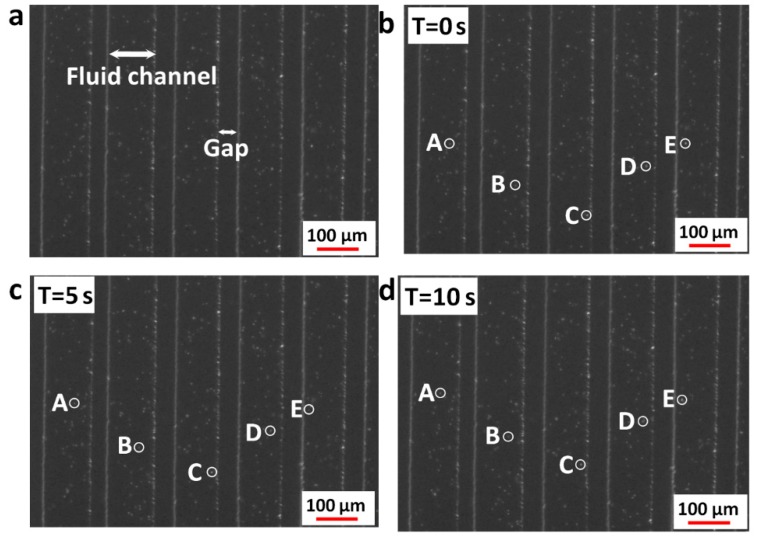
Sequential images of fluorescent particle movements of EOF. (**a**) Image instruction; (**b**) particle status at 0 s; (**c**) particle status at 5 s; (**d**) particle status at 10 s.

Figure 5 shows the sequential images of fluorescent particle movements of EOF. The applied voltage between electrodes is 10 V in this figure. Figure 5b–d show particle status at different time. Those particles with the same letters A~E marked in each figure are the same particles at different times. The times in Figure 5b–d are 0 s, 5 s and 10 s, respectively. In the real calculation process, we calculated 15 particles at different voltages with each 5 channels (60 particles in all, 3 particles in every channel), and the average velocity of all these particles are used as the fluid velocity.

**Figure 6:**
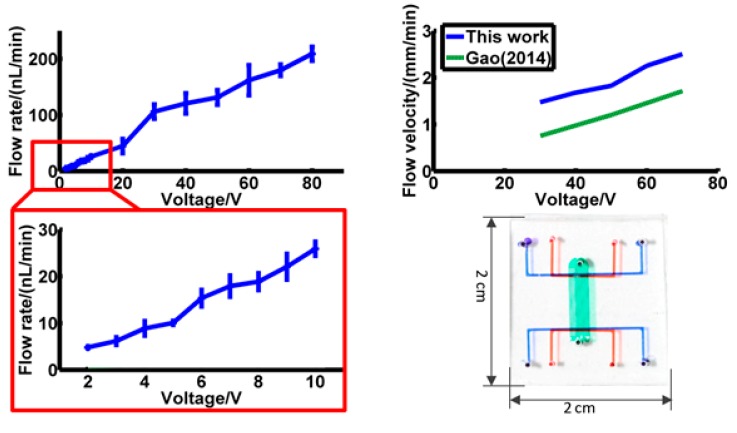
Flow rate at different voltage. (**a**) flow rate from 2 V to 80 V; (**b**) flow rate from 2 V to 10 V; (**c**) comparison of this work with Gao’s work [1] with similar dimensions; (**d**) size of this pump.

Flow rate was then calculated with fluid channel width 116 μm, height 31 μm and channel number 20. Results are shown in Figure 6, and actual channel dimensions are shown in Figure 6. In Figure 6a, the flow rate of 2 V to 80 V is shown, and flow rate from 2 V to 10 V is magnified and shown in Figure 6b. The flow rate is 5.69 nL/min at 2 V, and 248.18 nL/min at 80 V. The lowest driving voltage is 2 V.

The results show a linear trend between the flow rate and voltage. The error source might be the choice of particles during calculation. In electroosmotic flow, fluid velocity is higher near the solid edge, and lower in the middle of the channel. We tried to choose these 3 fluorescent particles in every channel as evenly as possible, however errors might exist.

During the early stage of this experiment, a 30 μm-thick top membrane was tested, and the membrane was too easy to tear while removing the pattern from its base, because of the existence of the fluid channel. So, we chose a 43 μm-thick top membrane instead. Thinner top and bottom membranes might make a higher electric field in the fluid channel with the same voltage, but are hard to make into a successful pump. Also, even two membranes are with different thickness, the homogeneity of the electric field in fluid channels would stay the same.

Placing electrodes on both sides of the fluid channels can increase the electric field strength and uniformity. To verify the merit of this structure, another experiment was performed with only one electrode layer, with the same design parameters and the same data calculation process. Results show that at 10 V, one electrode structure has a fluid velocity of 0.053 mm/min, which is only 1/8 of the velocity of the two electrode-layer structures. Thus, two-layer design makes the pumping more efficient.

Compared with 2-dimentional single channel EOF pumps [1], the flow rate of this pump has apparently increased. Gao previously designed a 2-D structure in 2014 [1]. With similar design parameters and material, the flow velocity of Gao’s pump is 1.32 mm/min at 50 V. In this work, this result is 2.18 mm/min. It is certain that, even though the flow velocity of these two pumps are similar, the flow rate of this pump is more than 100 times higher than in Gao’s design [1], due to fluid channel dimension and number. What’s more, in Gao’s design, the pump volume is 0.9 cm^3^ (1.5 cm × 3 cm × 2 mm). To get a similar flow rate by parallel connection 100 of that pump, the volume would be 90 cm^3^. The size of pump in this work is only 2 cm^3^ (2 cm × 2 cm × 0.5 cm), about 1/50 of that size.

The largest advantages of this pump are its high flow rate and low pumping voltage. By making a thin membrane between fluid channels and electrodes, the pumping voltage can be as low as 2 V, pretty low in noncontact electrode EOF pumps. By using parallel fluid channel design, flow rate is significantly increased. Also, this is an expandable structure. For some former EOF pumps [10,13,18], to increase flow rate by doubling fluid channel number, the whole pump volume is also doubled. For this pump, all fluid channels share the same pairs of electrodes, and to expand the fluid channel number from 20 to 40, only a 15% volume increase is needed. In the contact electrode structure, the fabrication process is always complicated, and expensive electrode material, like platinum, is used. In this noncontact design, room temperature liquid metal is cheap, and can be injected into channels to form electrodes, greatly simplifying the fabrication process. Noncontact electrodes also prevent sample contamination problems, joule heat problems, and strong electrophoresis, and prolong the pump’s lifespan.

There are still limitations to this pump. Even though the flow rate has been increased over 100 times, it is still not enough. To get a 5 mL/min flow rate, 2000 V voltage is needed. To increase the flow rate, a shorter distance between electrodes on the same layer could be used.

## 4. Conclusions

In this study, we proposed and tested a new 3-dimentioanl low voltage EOF pump. A five-layer structure is fabricated in this pump, including one fluid channel layer, two electrode channel layers and two thin membranes between them. The fluid channel layer contains 20 parallel fluid channels, lying in the middle of the two electrode layers. The distance between fluid channels and both electrode channels is under 45 μm (43 μm and 16 μm). Room temperature liquid gallium-based allay was used as the electrodes. A fluid flow rate of 248.18 nL/min was achieved at 80 V, and the lowest driving voltage is 2 V. In the future, more work would be done to lower the driving voltage and apply it in biological analysis and drug delivery systems.
